# Significant Impacts of Both Total Amount and Availability of Heavy Metals on the Functions and Assembly of Soil Microbial Communities in Different Land Use Patterns

**DOI:** 10.3389/fmicb.2019.02293

**Published:** 2019-10-04

**Authors:** Zhen Zhen, Sibo Wang, Shuwen Luo, Lei Ren, Yanqiu Liang, Rongchao Yang, Yongtao Li, Yueqin Zhang, Songqiang Deng, Lina Zou, Zhong Lin, Dayi Zhang

**Affiliations:** ^1^Agriculture College, Guangdong Ocean University, Zhanjiang, China; ^2^Faculty of Chemistry and Environmental Sciences, Guangdong Ocean University, Zhanjiang, China; ^3^College of Natural Resources and Environment, South China Agricultural University, Guangzhou, China; ^4^Research Institute for Environmental Innovation (Tsinghua), Suzhou, China; ^5^School of Environment, Tsinghua University, Beijing, China

**Keywords:** land use, heavy metals, heavy metal availability, soil bacterial community, ecological function

## Abstract

Land use change alters the accumulation of heavy metals (HMs) in soils and might have significant influence on the assembly and functions of soil microbial community. Although numerous studies have discussed the impacts of either total amounts or availability of metals on soil microbes in land change, there is still limited understanding on which one is more critical. In the present study, soils from three land use types (forest, mining field, and operating factory) located in Shaoguan city (Guangdong Province, China) were collected to investigate the impacts of soil HMs on soil enzyme activities and bacterial community structures. Mining activities remarkably increased the concentrations of HMs in soils, and land use patterns changed soil properties and nutrition level. Soil pH, total and available HMs (Cu, Pb, Zn, and Cd) and organic matters (SOM) were identified as the key influential factors shaping soil ecological functions (soil enzyme activities) and community assembly (bacterial community composition), explained by HMs accumulation and soil acidification caused by human activities. In addition, total amount and availability of some metals (Zn, Pb, Cu, and Cd) showed similar and significant effects on soil bacterial communities. Our findings provide new clues for reassessing the environmental risks of HMs in soils with different land use.

## Introduction

Land use change is one of the most important environmental changes affecting the biodiversity and function of ecosystems (Ferreira et al., 2016). Caused by the intensive anthropogenic activities and urbanization, land use change has a significant influence on soil quality (Pielke, 2005; Xia et al., 2011). Consequently, there are numerous environmental problems accompanying with land use change, and soil contamination by heavy metals (HMs) has become a major one (Huang Y. et al., 2018; Pacwa-Plociniczak et al., 2018). Previous studies suggest the significant influence of land use pattern on the accumulation of HMs in soils (Liu et al., 2016; Trujillo-González et al., 2016). Inappropriate land use is reported to result in the excessive input of HMs and affect soil biological properties (Papa et al., 2010; de Quadros et al., 2016). Owing to the significant toxicity and non-biodegradable property, HMs have long-term effects on soil ecosystems and public health (Liu et al., 2014; Islam et al., 2015a). HMs accumulation under different types of land use and their detrimental effects on soil quality have attracted increasing attention (Zhao et al., 2007; Jiao et al., 2010; Ren et al., 2015).

The influence of land use change on soil physical and chemical properties has been well studied (Islam and Weil, 2000; Giertz et al., 2005; Cherubin et al., 2015) and is reported to vary across land use types (de Quadros et al., 2016; Wu et al., 2017). The conversion of land use can alter soil quality by changing soil organic matters (SOM), cycles of carbon and nitrogen, texture and pH (Zeng et al., 2009; Moghimian et al., 2017). Land use change significantly decreased the contents of silt and clay, nitrogen, fulvic- and labile-carbon in tropical forest ecosystems (Islam and Weil, 2000). The change of shrub land to arable land and nursery garden significantly increased SOM, cation exchange capacity (CEC), total nitrogen and available nutrients (N, P, K) (Qi et al., 2018). The effects of forest converting to agricultural land on soil properties were also widely discussed (Murty et al., 2002; Bruun et al., 2015), and the conversion of forest to cropland led to the loss of soil carbon stocks (Perrin et al., 2014; Reza et al., 2018). However, the influence of land use change from forest to industrial fields on soil quality and ecological function, particularly in case of HMs contamination, is barely studied and how HMs contamination in land change drives the microbial community structure is still unknown.

Soil enzyme activities are increasingly recognized as crucial indicators for soil ecological functions (Huang et al., 2017; Wu et al., 2017). Soil physicochemical properties, e.g., pH, SOM, CEC, and humus, could influence soil micro-environment and ecological functions (Chodak et al., 2013; Huang et al., 2017). In addition, excessive HMs contamination affected the structure and diversity of soil microbial communities and inhibited soil enzyme activities, consequently damaging microbial metabolic abilities, decreasing soil ecological functions and weakening soil resistance to other disturbances (Duan et al., 2018). Accordingly, soil microbial community structure was significantly affected by both soil properties and HMs, such as pH, SOM, Cd, Pb, and Zn (Guo et al., 2017). They were reported to show significant influence on the changes of bacterial community structure and diversity in a previous study, and Cr and Cd were the major factors related to the change in bacterial assembly (Zhang C. et al., 2016). The integral effects of HMs and soil properties on soil enzyme activities were unraveled, and the main driving force was SOM (Xian et al., 2015).

Nevertheless, there is still questionable which shows more significant impacts on soil microbial community, total amount or availability of HMs. Although most of previous studies focused on the total amount of HMs to assess the environmental risks (Bai et al., 2011; Gu et al., 2014), recent studies suggested the strong correlation between HMs toxicity and availability (Kim et al., 2015; Xiao et al., 2015; Zhang et al., 2017). Available HMs predicted metal transfer from soils to crops better than HMs total amount (Amini et al., 2005). As land use could influence both total amount and availability of HMs by human activities (Karim et al., 2014) and soil properties (Song et al., 2009), it is of great importance to distinguish the contribution of total amount and availability of HMs on soil microbial assembly. HMs concentrations, soil properties and land use pattern were reported to integrally cause high human health risks (Zhao et al., 2012). Most of the studies focused on the distribution and accumulation of total HMs (Xia et al., 2011; Jiao et al., 2014; Islam et al., 2015b; Wang and Xu, 2015), and little information is available on the change in HMs availability in different land use patterns. Thus, the effects of total or available HMs, accompanying with soil physicochemical properties, on bacterial community structure and enzyme activities need to be distinguished in different land use patterns.

In the present study, we hypothesized that both total amount and availability of HMs could significantly affect soil microbial assembly (bacterial composition) and ecological function (soil enzyme activities) through the change of land use, and they should be simultaneously considered for ecological risk assessment. Three types of land use including operating factories, mining fields and forest fields from Shaoguan city (Guangdong Province, China) were investigated *via* 16S rRNA gene sequencing and diffusive gradient in thin films (DGT). This study aimed to: (1) evaluate the impacts of land use change on soil properties, bacterial community composition and enzyme activities; (2) identify key influential environmental factors shaping soil microbial community and ecological function; (3) compare and evaluate the impacts of total amount and availability of HMs. This study unveils the equal importance of total amount and availability of different HMs in shaping microbial community and ecological function, and gives clues for reassessing the environmental risks of HMs through land use change.

## Materials and Methods

### Study Area and Soil Collection

**FIGURE 1:**
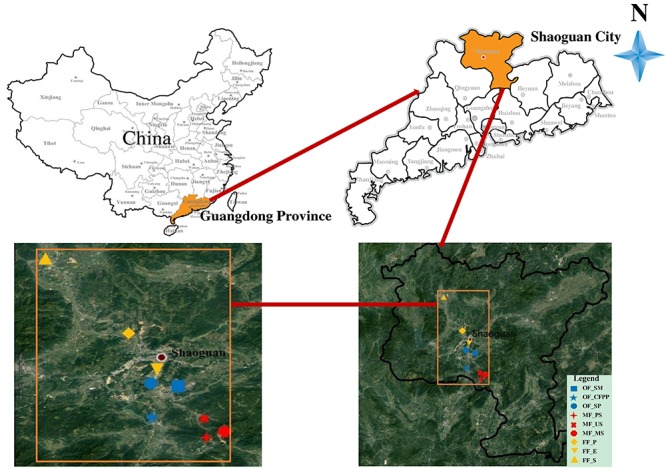
Distribution of sampling sites (*n* = 9) in study area.

Soils were collected in July 2016 at Shaoguan city (locations see Figure 1), which is a prefecture-level city in the northern Guangdong Province, China. The average annual rainfall in study area located in the subtropical region is 1457 mm and the average annual temperature is 20°C. Latosolic red soils and red soils are the main soil types. Mining activities have lasted several decades in this region and there are still many lignite-fired power plants, smelters and steel mills. The three land use types included operating factories (OF: steel mill, OF_SM; coal-fired power plant, OF_CFPP; smelting plant, OF_SP), mining fields (MF: paddy soils, MF_PS; upland soils, MF_US; mining soils, MF_MS) and forest fields (FF: pine, FF_P; eucalyptus, FF_E; shrubs, FF_S). Soil samples of the nine sites were collected at 0–20 cm depth with five replicates randomly in a 100 m^2^ area and their coordinates were determined using a portable GPS unit (Supplementary Table S1). All samples were collected using a bamboo spade, transported to the laboratory in polytetrafluoroethylene (PTFE) bags, and then separated into two parts. One part was frozen in liquid nitrogen immediately and stored in a −80°C fridge for biological analysis. The other part was kept in the ice box and stored at 4°C for the analysis of HMs and soil physicochemical properties.

### Soil Properties Analysis

Soil pH was measured with a pH meter using a soil-to-water ratio of 1:2.5 with 0.01 M CaCl_2_ and SOM was determined by H_2_SO_4_-K_2_Cr_2_O_7_ oxidation method (Hu et al., 2015; Deng et al., 2018). Soil CEC was measured by the ammonium acetate method (Choo and Bai, 2016). Soil humus (fulvic acids, humic acids, and humin) was alkaline extracted and determined following the NAGOYA methods (Kuwatsuka et al., 1992). Soil total nitrogen (TN) was determined by Kjeldahl method (Bremner, 1960). After digestion with perchloric acid and hydrofluoric acid, soil total phosphorus (TP) and potassium (TK) were measured by colorimetric assay (ammonium molybdate) and atomic absorption spectrometry (AAS), respectively (Humayun and Clayton, 1995; Cheng et al., 2016). For analysis of total HMs, soils were digested by a concentrated acid mixture and the HM contents were quantified by Inductively Coupled Plasma-Atomic Emission Spectrometry (ICP-AES, Prodigy XP LEEMAN, China) (Wu et al., 2017). Available HMs were determined by diffusive gradient in thin-films (DGT) using a C-LSLM loaded DGT device following manufacturer’s instruction (DGT Research Limited Corporation) (Yao et al., 2016). The average recovery of Cu, Pb, Zn, Cr, Cd, and Ni was 95.42, 102.54, 95.57, 92.34, 104.72, and 93.17%, respectively.

### Soil Microbial Biomass and Enzyme Activity Analysis

Soil microbial biomass carbon (MBC) and nitrogen (MBN) were determined by the fumigation-extraction method (Brookes et al., 1985; Vance et al., 1987). Soil microbial activity was measured by basal respiration (Insam, 1990). Soil urease, catalase, invertase, acid phosphatase, and dehydrogenase activities were determined by the phenol-sodium hypochlorite colorimetric method, potassium permanganate titration method, 3,5-dinitrosalicylate method, disodium phenyl phosphate colorimetric method and triphenyl tetrazolium chloride (TTC) colorimetric method, respectively, according to previous studies (Schinner and Vonmersi, 1990; Garcia-Gil et al., 2000).

For urease activity, 5.0 g of soils (dry weight) were mixed with 1.0 mL of toluene for 15 min, and incubated for 24 h at 37°C with 10 mL of urea solution (10%) and 20 mL of citrate buffer (pH = 6.7). Then, 3 mL of the supernatant was mixed with 20 mL of distilled water, 4 mL of sodium phenol and 3 mL of sodium hypochlorite for 20 min, and spectrophotometrically analyzed at 578 nm.

The 2.0 g of soils (dry weight) were added with 40 mL of distilled water and 5 mL of hydrogen peroxide (0.3%) to determine catalase activity. After 20 min shaking, the mixture was added with 1 mL of saturated potassium aluminum sulfate dodecahydrate, filtered, and mixed with 5 mL of sulfuric acid (1.5 mol/L). Then, 25 mL of the supernatant was titrated with potassium permanganate (0.02 mol/L) to detect the residual hydrogen peroxide.

Invertase activity was measured by mixing 5.0 g of soils (dry weight), 15 mL of sucrose solution (8%), 5 mL of phosphate buffer (pH = 5.5) and 5 drops of toluene. The reaction system was incubated for 24 h at 37°C, and 1 mL of filtrate was added with 3 mL of 3,5-dinitrosalicylic acid for 5 min in boiling water, and spectrophotometrically analyzed at 508 nm.

For acid phosphatase activity, 5.0 g of soils (dry weight) were mixed with 2.5 mL of toluene and gently shaken for 15 min. After adding 10 mL of disodium phenylphosphate solution and 10 mL of acetate buffer (pH = 5.0), the system was further incubated for 24 h at 37°C. The mixture was then diluted with ultrapure water (38°C) to 100 mL and filtrated. The 3 mL of the supernatant was mixed with 5 mL of boric acid buffer and 4 drops of Gibbs reagent and reacted for 30 min to measure released phenol colorimetrically at 660 nm.

Dehydrogenase activity was measured by mixing 5.0 g of soils (dry weight) with 5 mL of TTC (0.5%), incubating for 24 h at 37°C, and extracted with 40 mL methanol after 1 h shaking. The absorbance of the supernatant was determined at 485 nm.

### Soil Bacterial Community Structure

Soil DNA was extracted using PowerSoil DNA extraction kit (MoBio, United States) according to manufacturer’s instructions. DNA concentration was determined using an ND-2000 UV-Vis spectrophotometer (NanoDrop Technologies, United States). The hypervariable V4 region of 16S rRNA gene was subsequently amplified by polymerase chain reaction (PCR) using the primer pair of 515F (5′-GTGCCAGCMGCCGCGGTAA-3′) and 806R (5′-GGACTACVSGGGTATCTAAT-3′) with barcode (Sengupta and Dick, 2017). Purified PCR amplicons were sequenced by an Illumina HiSeq2500 platform (Novogene, China). Reads were chosen after the quality filtering, and the reads were discarded if the barcodes were uncorrectable. Chimeras were removed and the sequences with high quality were clustered into different operational taxonomic units (OTUs) based on 97% similarity using Uparse^1^. The representative OTU sequences were chosen for taxonomical classification using QIIME pipeline and Ribosomal Database Project (RDP) (Xu et al., 2017). The sequences were archived at Genbank (BioSample accession: SAMN12734515; BioProject ID, PRJNA565100).

### Data Analyses

All data were plotted using Origin (version 8.1). Alpha-diversity (Chao1 and Shannon) was used to estimate the complexity of bacterial community in different samples using QIIME software^2^. Unweighted Pair-group Method with Arithmetic means (UPGMA) was performed as the hierarchical clustering method interpreting the metric distance matrix using average linkage and cluster of bacterial genera by QIIME software^3^. The major bacterial lineages in total sequences (top 10) exhibited the heatmap analysis and species classification tree among different samples, using R software and MEGAN, respectively^4^. Before redundancy analysis (RDA) or canonical correlation analysis (CCA), detrended correspondence analysis (DCA) was conducted and the length of gradient determined the following procedure (<3: RDA; >4: CCA; 3–4: RDA or CCA). According to the length of gradient, RDA was conducted to investigate the impacts of environmental factors on soil enzyme activities, and CCA was applied to explore the effects of environmental variables on bacterial community structure using CANOCO 5.0. One-way ANOVA and Duncan’s test were performed to assess the statistically significant differences of HMs between samples within the same site and between samples across sites of different land use pattern by using SPSS 21.0. As for soil properties, One-way ANOVA and Duncan’s test were only performed to assess the statistically significant differences between samples across sites of different land use pattern.

## Results

### Soil Physicochemical Properties

Soil physicochemical properties across different land use patterns are listed in Table 1. Significant soil acidification was observed in MF (average pH = 3.75) and OF (average pH = 5.40), whereas FF soils were only slightly acidic with pH ranging from 5.83–6.84. Except for TK content, other soil physicochemical properties showed moderate coefficient of variation (*cov*, from 22.5 to 30.1%). In MF soils, SOM (31.96 g/kg, average from the different sites of the same land use pattern, the same as follows), humus (20.21 g/kg, CEC (13.60 mol/kg), TN (2.47 g/kg), and TP (13.60 mg/kg) were relatively higher than those at OF and MF sites. TK content had huge variation (*cov* = 44.7%) across sites and did not show remarkable difference between soils in FF (16.06 mg/kg), OF (10.47 mg/kg), and MF (21.64 mg/kg, *p* > 0.05).

**TABLE 1:** Soil physicochemical properties across different land use patterns.

**Samples**	**pH**	**SOM (g/kg)**	**Humus (g/kg)**	**CEC (mol/kg)**	**Total N (g/kg)**	**Total P (mg/kg)**	**Total K (mg/kg)**
OF_SM	5.89 ± 0.05^b^	18.55 ± 0.89^d^	10.78 ± 0.13^d^	10.89 ± 0.49^b^	1.39 ± 0.01^e^	0.52 ± 0.02^c^	6.30 ± 0.12^f^
OF_CFPP	5.14 ± 0.04^c^	20.28 ± 0.12^cd^	11.21 ± 0.30^d^	8.46 ± 0.03^d^	1.65 ± 0.02^d^	0.87 ± 0.29^b^	17.24 ± 0.14^c^
OF_SP	5.19 ± 0.03^c^	21.13 ± 0.06^c^	11.74 ± 0.14^d^	7.47 ± 0.06^e^	1.84 ± 0.03^c^	0.63 ± 0.02^c^	7.88 ± 0.17^e^
MF_PS	3.87 ± 0.01^d^	20.16 ± 1.41^cd^	12.27 ± 0.36^d^	7.11 ± 0.18^e^	1.16 ± 0.01^e^	1.02 ± 0.02^b^	18.32 ± 0.31^b^
MF_US	4.17 ± 0.01^d^	22.14 ± 0.55^c^	14.33 ± 0.11^c^	5.71 ± 0.08^f^	1.72 ± 0.01^cd^	0.85 ± 0.01^b^	17.04 ± 0.34^c^
MF_MS	3.22 ± 0.01^e^	13.24 ± 0.79^e^	6.04 ± 0.26^e^	4.45 ± 0.04^g^	0.50 ± 0.03^f^	0.52 ± 0.02^c^	29.55 ± 0.38^a^
FF_P	5.83 ± 0.01^b^	28.40 ± 0.45^b^	18.47 ± 0.39^b^	15.99 ± 0.40^a^	2.31 ± 0.02^b^	0.92 ± 0.01^b^	19.70 ± 0.32^b^
FF_E	6.51 ± 0.03^a^	28.45 ± 0.68^b^	19.52 ± 0.78^b^	9.05 ± 0.15^c^	1.92 ± 0.02^c^	0.84 ± 0.01^b^	18.72 ± 0.24^b^
FF_S	6.84 ± 0.04^a^	39.03 ± 3.08^a^	22.64 ± 0.03^a^	15.77 ± 0.05^a^	3.19 ± 0.02^a^	1.24 ± 0.03^a^	9.75 ± 0.18^d^

**Data are mean ± standard deviation (SD) obtained from five replicates. Data with different lower-case letters refer to significant differences (p < 0.05) among the samples and the same letter indicates no significant difference.**

### Total Amount and Availability of Heavy Metals

**FIGURE 2:**
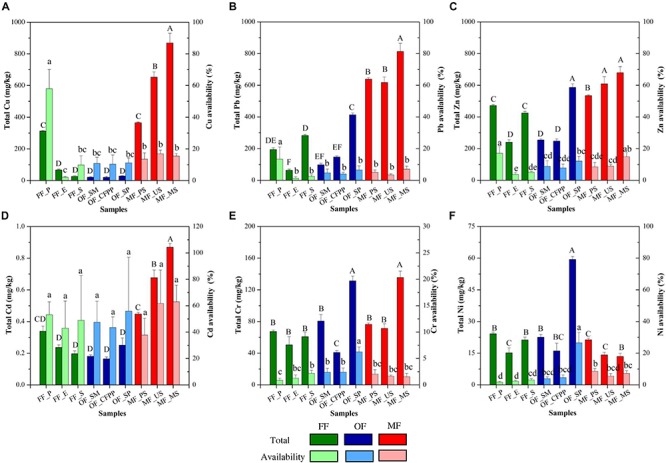
Total amount and availability of HMs across different land use patterns. **(A)** Cu, **(B)** Pb, **(C)** Zn, **(D)** Cd, **(E)** Cr, and **(F)** Ni. Data are mean ± standard deviation (SD) obtained from five replicates. Bars with different lower-case letters refer to significant differences (*p* < 0.05) among the samples and the same letter indicates no significant difference.

Total amount and availability of HMs in soils are illustrated in Figure 2. Generally, HMs total amount followed the order: Zn (449.84 mg/kg) > Pb (363.04 mg/kg) > Cu (262.13 mg/kg) > Cr (79.38 mg/kg) > Ni (23.06 mg/kg) > Cd (0.37 mg/kg). MF soils had significantly higher total amount of Cu (628.67 mg/kg, Figure 2A), Pb (689.44 mg/kg, Figure 2B), Zn (607.80 mg/kg, Figure 2C), and Cd (0.66 mg/kg, Figure 2D) than those in FF and OF (*p* < 0.05). The total amount of Cr and Ni was similar across different land use patterns (*p* > 0.05).

Available HMs followed the order: Zn (39.00 mg/kg) > Cu (30.96 mg/kg) > Pb (15.32 mg/kg) > Cr (1.59 mg/kg) > Ni (1.20 mg/kg) > Cd (0.17 mg/kg). In FF soils, available Zn was the highest (40.53 mg/kg, Supplementary Figure S1C), followed by Cu (38.09 mg/kg, Supplementary Figure S1A), Pb (16.53 mg/kg, Supplementary Figure S1B), Cr (1.64 mg/kg, Supplementary Figure S1E), Ni (1.36 mg/kg, Supplementary Figure S1F), and Cd (0.19 mg/kg, Supplementary Figure S1D). Available metals in OF soils were similarly to those in FF soils (*p* > 0.05), and available Cu, Pb, Zn, and Cd were significantly higher in MF soils than FF and OF soils (*p* < 0.05). Accordingly, Cd availability was the highest across all sites (47.3%, Figure 2), followed by Cu (11.8%), Zn (8.7%), Ni (5.2%), Pb (4.2%), and Cr (2.0%). FF had the highest availability of Cu (58.2%), Pb (11.2%), and Zn (19.3%), and the highest availability of Cd was found in MF soils (61.9%). OF soils had the highest availability of Cr (6.1%) and Ni (22.8%).

### Soil Biochemical Properties and Influential Factors

**FIGURE 3:**
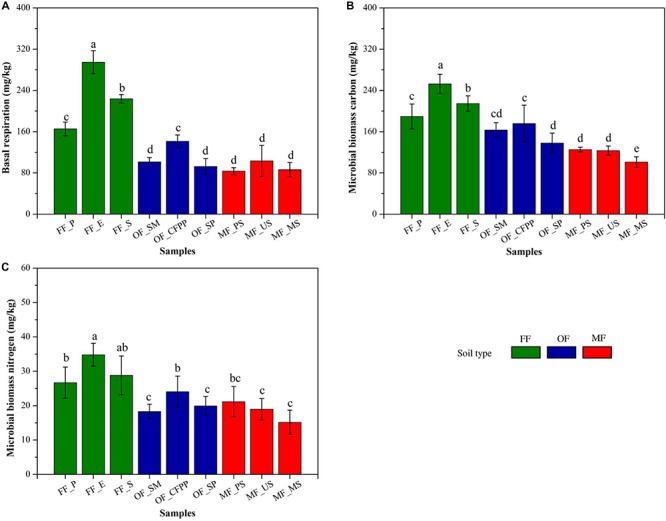
Soil biochemical properties across different land use patterns. **(A)** Soil basal respiration, **(B)** microbial biomass carbon and **(C)** microbial biomass nitrogen. Data are mean ± standard deviation (SD) obtained from five replicates. Bars with different lower-case letters refer to significant differences (*p* < 0.05) among the samples and the same letter indicates no significant difference.

**FIGURE 4:**
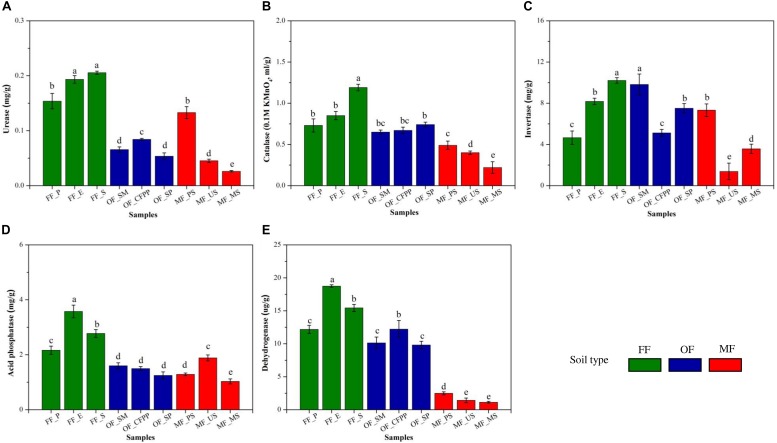
Soil enzyme activities across different land use patterns. **(A)** Urease, **(B)** catalase, **(C)** invertase, **(D)** acid phosphatase, and **(E)** dehydrogenase. Data are mean ± standard deviation (SD) obtained from five replicates. Bars with different lower-case letters refer to significant differences (*p* < 0.05) among the samples and the same letter indicates no significant difference.

Soil basal respiration, MBC and MBN are illustrated in Figure 3. FF soils had higher soil basal respiration (228.16 mg/kg, Figure 3A), MBC (218.81 mg/kg, Figure 3B), and MBN (30.20 mg/kg, Figure 3C) than other soils (*p* < 0.05), and there was no significant difference between soils at MF and OF sites (*p* > 0.05). Figure 4 shows soil enzyme activities representing soil ecological functions. It was obvious that the activities of urease (0.18 mg/g, Figure 4A), catalase (0.92 mL/g, Figure 4B), and acid phosphatase (2.84 mg/g, Figure 4D) were the highest in FF soils. On the contrast, invertase activity varied across land use patterns with no significant difference (*cov* = 21.2%, *p* > 0.05, Figure 4C) and dehydrogenase activity was lowest in MF (1.67 μg/g) comparing to that in FF (15.45 μg/g) and OF (10.72 μg/g, Figure 4E).

**FIGURE 5:**
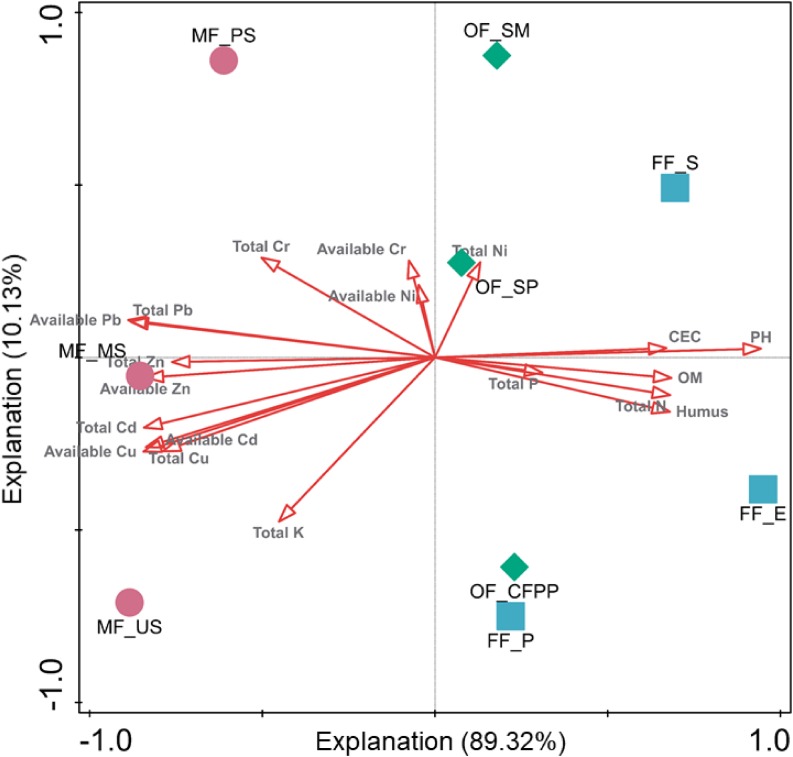
Redundancy analysis (RDA) of total HMs, available HMs, soil properties and soil enzyme activities. Arrows indicate the direction and magnitude of environmental factors associated with soil enzyme activities.

To evaluate the effects of soil properties, total HMs and available HMs on soil enzyme activities, RDA was performed and the first two axes explained 89.32 and 10.13% of the total variance, respectively (Figure 5). The first axis was driven by soil properties (pH, CEC, SOM, TN, and humus), total Cd/Pb/Cu/Zn and available Cd/Pb/Cu/Zn, whereas the second axis was driven by TK, total Ni/Cr and available Ni/Cr. It is worth noting that soils at different sites were segregated, indicating land use change exhibited different driven forces and led to distinct soil enzyme activities.

### Bacterial Community Structure and Influential Factors

A total number of 252,085 high quality reads for bacterial 16S rRNA gene were obtained from all the soil samples. The average reads of soils in OF, MF, and FF were 27,624, 22,210, and 34,194, respectively (Supplementary Table S2). The OTU numbers were 2,736, 2,161, and 3,094 for OF, MF, and FF soils. For α-diversity indices, Shannon index followed the order of FF (8.13) > OF (6.90) > MF (5.86) and Chao 1 was also the lowest in MF (4,341), suggesting soils in MF had the lowest bacterial α-diversity.

**FIGURE 6:**
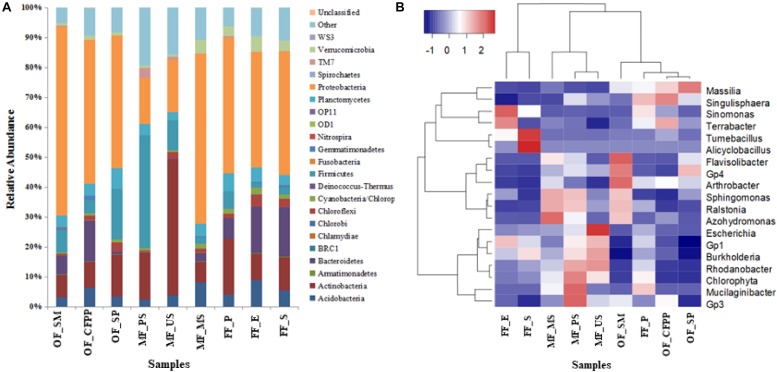
Relative abundance (%) of dominant bacterial phyla **(A)** and heatmap of bacterial community at the genus level (abundance > 0.5%) based on Bray-Curtis distance **(B)**.

The taxonomic information at the phylum level across different land use patterns are shown in Figure 6A. Among all the identified bacterial phyla, *Proteobacteria* (15.37–61.69%) were most frequently detected, followed by *Actinobacteria* (6.89–44.04%), *Firmicutes* (1.39–37.44%), *Bacteroidetes* (0.35–15.75%), *Acidobacteria* (2.36–8.65%), *Planctomycetes* (2.57–6.58%), *Verrucomicrobia* (0.72–4.95%), *Chloroflexi* (0.54–3.98%), and *Cyanobacteria* (0.47–2.35%). In FF soils, *Bacteroidetes*, *Chloroflexi*, *Verrucomicrobia, Acidobacteria*, and *Cyanobacteria* were abundant, whereas *Firmicutes* had the lowest abundance. The relative abundance of *Firmicutes* and *Actinobacteria* was higher in MF soils. No significant difference was found in the relative abundance of *Planctomycetes* among all soil samples.

At the genus level, microbial composition differed substantially across different land use patterns. In FF soils, dominant bacterial genera included *Massilia*, *Escherichia*, *Mucilaginibacter*, *Flavisolibacter*, *Singulisphaera*, *Terrabacter*, and *Sinomonas*, accounting for 34.30% of the whole community. *Massilia*, *Flavisolibacter*, *Singulisphaera*, and *Terrabacter* were dominant in OF soils, whereas the most abundant bacterial genera in MF soils were assigned to *Escherichia*, *Tumebacillus*, *Sinomonas, Alicyclobacillus*, and *Terrabacter*. According to the heatmap of bacterial community (Figure 6B), soils in MF differed from those in OF and FF.

**FIGURE 7:**
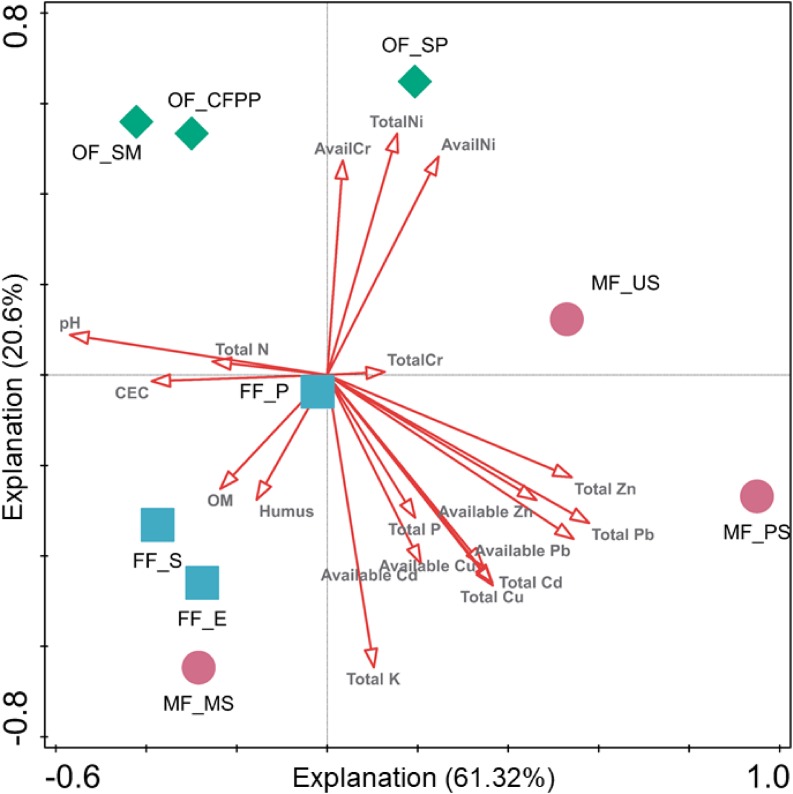
Canonical correlation analysis (CCA) of bacterial genera and environmental factors. Arrows indicate the direction and magnitude of environmental factors associated with bacterial community structure.

To further evaluate the influence of environmental variables on soil microbial community composition across different land use patterns, CCA of bacterial genera and environmental factors was performed and the eigenvalues of the first two axes explained 61.32 and 20.60% of the total variance (Figure 7). The first axis was driven by pH, CEC, TN, total Zn/Pb and available Zn/Pb, while the second axis was driven by TK, TP, total Ni/Cd/Cu and available Ni/Cd/Cu/Cr. Again, microbial communities in soils from different land use types were segregated by these environmental variables. Taking both soil enzyme activities and microbial community composition together, the core factors affecting soil ecological functions and microbial assembly during the land use change from forests to mining or industrial fields included soil properties (pH, CEC, and TK), total Zn/Pb/Cu/Cd and available Zn/Pb/Cu/Cd.

## Discussion

Land use change has dramatically altered the characters of earth surface and led to a series of environmental problems. Particularly, mining and industrial activities destroyed many forests and showed negative impacts on soil ecosystems (Lauber et al., 2008; Wu et al., 2017). Land use change from forests to mining and industrial fields is relatively a short-term process compared to other geochemical processes, usually causing the changes in soil properties and HMs accumulation which play key roles in shaping soil ecological functions and microbial assembly (Tischer et al., 2015; Jiang et al., 2016; Zhang C. et al., 2016). In the present study, land use change from FF to MF or OF was comprehensively investigated to explore the mechanisms shaping soil enzyme activities and microbial communities. Our findings suggested significant change in soil properties through land use change, which might further affect soil microbial assembly and ecological functions. The pH in MF and OF soils was 1–2 units lower than that in FF soils (Table 1), representing that mining and industrial activities acidified soil pH by dumping wastes and discharging wastewater. This conclusion is supported by some previous studies, which reported that mine and industrial wastes released from mining activities polluted nearby soils and water, degraded soil quality and consequently damaged the ecological functions and microbial assembly during the land use change process (Liao et al., 2005; Xue et al., 2017). Additionally, the total amounts of HMs increased in MF and OF soils, whereas the availability of Cu, Pb and Zn and Cd rose through land use change from FF to MF or OF, potentially correlated with the change in soil properties, e.g., decreases of SOM, humus, CEC, TN, and TP.

The total amount of Cu, Pb, and Zn in most FF soils were above the Standard of Soil Quality Assessment for Exhibition Sites in China (63, 140, and 200 mg/kg, respectively) (HJ350-2007), whereas Cd, Cr and Ni were within acceptable level. These results indicated a high background of Cu, Pb, and Zn in soils in the present study. In MF soils, the total amounts of Cu, Pb, and Zn were 4.66, 3.83, and 1.60 times higher than FF, whereas there was unexpectedly no significant difference in total HMs between FF and OF soils (Figure 1), hinting the negligible effects of industrial activities on soil HMs total amount. Cu, Pb, and Zn were the major constituents of ore minerals and could be discharged into surrounding soils *via* atmospheric deposition accompanying with the mining activities (Lu et al., 2015). In the present study, MF soils were seriously polluted by HMs because the surrounding mining activities have lasted for decades, consistent with previous studies (Wang X.Q. et al., 2016; Leung et al., 2017). Similar results were reported in Liang’s work that HMs contents were related to the anthropogenic source across different land use patterns (Liang et al., 2017a). In present study, the major origins of HMs in MF soils were also natural sources, explained by the high-level of HMs content in upland soils (MF_US) which was not affected by human activities. Accordingly, it is questionable to clean up OF soils owing to the high background. Instead, risk control is a more promising way to protect human health and food crop safety.

Mining activities and industrial factories had significant influences on the availability of HMs in soils, although the impacts were less remarkable than those on total HMs. Accordingly, the highest HMs availabilities were found in MF soils, including Cu, Zn, Pb, Cd, and Ni, and relative higher availabilities of Pb, Zn, Ni, and Cr were found in OF soils comparing to FF soils. Our results were consistent with previous studies that both the total and available fraction of HMs, including Cd, Cr, Cu, Ni, Pb, and Zn were significantly affected by mining associated activities (Maiz et al., 2000). It was documented that HMs availability is also affected by soil properties like pH, SOM, CEC, redox potential, clay content, available phosphorus, and Fe/Mn oxides (Antoniadis et al., 2008; Zeng et al., 2011; Pietrzykowski et al., 2014; Yu et al., 2016). Among them, soil pH is reported to be the dominant one with negative correlations with HMs availability in many studies (Bang and Hesterberg, 2004; Rafiq et al., 2014). Low soil pH decreases the negative charge on clay minerals, hydrated oxides and organic surfaces (Guo et al., 2018), consequently weakening the adsorption of metals and increasing metal availability (Huang L.M. et al., 2018). In addition, the synergistic effects of HMs and SOM are also crucial in altering metal availability. HMs accumulation in soils can lead to the loss of soil nutrients (Zhao et al., 2014), including SOM which can significantly affect HMs availability by forming insoluble precipitates (such as sulfide) and reducing the content of exchangeable HMs (Zang et al., 2015). SOM also has oxygen-containing functional groups, which release negative charges and restrict the exchange capacity of metal cations in soils, strengthening the HMs adsorption and fixation capacity of soils (Guo et al., 2017). Similar mechanism was reported that the availability of Cd, Cu, and Zn declined with the increasing SOM *via* physical adsorption, precipitation and surface complexation (Guo et al., 2017; Huang L.M. et al., 2018). In the present study, long-term mining activities continuously discharged metals into the soil, reduced soil pH *via* atmospheric deposition and acid mine waste emission, and led to SOM loss, explaining the increasing HMs availability.

Land use change is known to influence the microclimate and soil abiotic/biotic properties (Nazaries et al., 2015), directly affecting soil basal respiration and microbial biomass which are suggested as indicators of soil quality (Yao et al., 2000; Yang et al., 2006). Comparing to FF soils, lower soil basal respiration and microbial biomass in OF and MF soils suggested soil quality degradation, consistent with previous study that the change of forest to other land uses caused the carbon loss as forests can sequester carbon in aboveground biomass and SOM (Zhang Q. et al., 2016). Soil microbial biomass is also sensitive to HMs (Yao et al., 2003) and both basal respiration and microbial biomass are negatively correlated with HMs contents (Yang et al., 2006).

Soil enzyme activity is a biological indicator for soil ecological functions and is easily affected by soil physical, chemical and biological properties (Wang C.F. et al., 2016). In the present study, the activities of urease, catalase and acid phosphatase in OF and MF soils were relatively lower than those in FF soils and dehydrogenase activity in MF soils was significantly lower (Figure 5), consistent with previous studies that soil enzyme activities like urease, sucrase, catalase and invertase were restrained in HMs contaminated areas (Nie et al., 2018; Zhai et al., 2018). It might be attributed to the change in soil pH, SOM, and HMs through land use change. Soil pH directly affected the biochemical reaction rates of soil enzymes *via* affecting the amino acid functional groups essential for binding and catalysis and influencing the effective concentration of inhibitors or activators in soils (Dick et al., 2000). Moreover, different soil enzymes prefer distinct pH optimum, e.g., 6.5–7.0 or 8.8–9.0 for urease and 4.0–5.0 for acidic phosphatase, resulting in distinct profiles of soil enzyme activities under different pH conditions. SOM and humus might also play key roles in regulating soil enzyme activities, as they could form enzyme-humus material complexes which maintain enzyme activity and postpone enzyme decomposition (Burgos et al., 2002; Zhen et al., 2014). It is reported that soil enzyme activities increased with SOM content (Burgos et al., 2002). HMs are reported to reduce enzyme activities by interacting with the enzyme-substrate complex, hindering the active groups of enzymes, denaturing the enzyme directly, or inhibiting enzyme synthesis (Moreno et al., 2003; Tian et al., 2010). In the present study, the decrease of soil enzyme activities during the land use change process from FF to OF or MF was attributed to the synergistic effects of soil acidification (Table 1), decrease of SOM (Table 1) and increase of HMs (Figure 2).

Land use change from FF to OF or MF also significantly altered soil microbial assembly, as soil microbial diversity decreased and bacterial communities were clustered according to their land use pattern (Figures 6, 7 and Supplementary Table S2). Soil pH and HMs are the key driven force shaping the microbial assembly during the land use change process in this study. Soil pH is identified as the most important factor in structuring the soil microbial community by numerous studies (Deng et al., 2018; Jiang et al., 2019). Any variation in pH exerts pressure on single-celled organisms, as the intracellular pH of most microorganisms is usually within 1 pH unit of neutral (Kuang et al., 2013). Significant acidification in MF and OF soils therefore inevitably altered the microbial community composition. Long-term HMs accumulation is another explanation for this alteration. In MF soils with serious HMs contamination, *Flavisolibacter*, *Sphingomonas*, *Ralstonia*, *Rhodanobacter*, *Chlorophyta*, and *Gp3* were predominant, consistent with previous studies (Guo et al., 2017; Bonanno and Orlando-Bonaca, 2018; Wang et al., 2018). This pattern is attributed to their capability of tolerating HMs contamination and detoxifying HMs by biosorption, bioprecipitation, extracellular precipitation, and chelation (Ni et al., 2016). According to EIEES theory (Everything is everywhere, but the environment selects), soil microorganisms susceptible to abrupt exposure of toxins (e.g., HMs) faded, whereas resistant ones adapted and flourished (Feng et al., 2016; He et al., 2017), resulting in the distinct microbial community composition through land use change. It is worth noting that soil pH can alter the availability and mobility of HMs, and consequently change metal toxicity and affect the microbial community significantly. Long-term acidification and HMs accumulation during the land use change process in this study together decreased SOM and affected the microbial community composition, *via* inhibiting microbial diversity and activity (Figures 4, 6). SOM is often associated with abundant and diverse metabolic carbon sources, which may significantly change the conditions of microbial habitats and immobilize HMs *via* precipitation to indirectly ameliorate potential toxic effects of HMs, benefiting soil microorganisms (Wu et al., 2017). In the present study, the loss of SOM and humus across land use types to different extent directed the traits of microbial assembly. Overall, long-term niche-filtering driving by acidification and HMs accumulation weeded out the sensitive microbes and shaped the unique microbial assembly in MF and OF soils.

It is still questioned which HMs speciation, total amount or available portion, can better represent HM toxicity (Liang et al., 2017b; Cambier et al., 2019; Ogunlaja et al., 2019). Here, we found different order between HMs total amount (Zn > Pb > Cu > Cr > Ni > Cd) and availability (Cd > Cu > Zn > Ni > Pb > Cr). It was therefore of importance to distinguish their influence on soil ecological functions and microbial composition. From RDA and CCA results, HMs with high total amount and low availability (Zn: 534.75–679.45 mg/kg, 9.01–11.36%, Pb: 617.21–812.92 mg/kg, 4.92–5.23%, and Cu: 364.80–868.85 mg/kg, 12.88–15.39%) or low total amount and high availability (Cd: 0.44–0.87 mg/kg, 36.23–62.28%) had similar and significant impacts on soil enzyme activities and microbial assembly. Although either available or total HMs were reported to individually play important roles in shaping microbial community by previous studies (Zhao et al., 2012; Deng et al., 2018), our results for the first time claimed that both total amount and availability of HMs showed significant influence on soil ecological functions and microbial community across land use patterns, and they should be simultaneously considered for ecological risk assessment. Nevertheless, the mechanisms of HM total amount and availability affecting soil microbes might vary across sites, which requires further study.

## Conclusion

This study provided novel and comprehensive insights into the effects of HMs and soil physicochemical properties on enzyme activities and bacterial community in different land use patterns. Soil properties and contents of some HMs, e.g., Zn, Pb, Cu, and Cd, varied across land use types and resulted in unique traits on ecological functions and microbial assembly. Long-term acidification and HMs accumulation were identified as the main driven forces. Both total amount and availability of HMs showed significant impacts, suggesting that their importance needs to be emphasized during the process of environmental risk assessment. Our findings offered new clues for reassessing the environmental risks of HMs by considering both total amount and availability.

## Data Availability Statement

The datasets analyzed in this manuscript are not publicly available. Requests to access the datasets should be directed to zhangdayi@tsinghua.edu.cn.

## Author Contributions

DZ and ZL designed the study. ZZ and SL conducted the experiments and interpreted the data. ZZ, SW, LR, YQL, RY, and SD contributed to the data analysis. ZZ, ZL, YTL, YZ, and DZ wrote the manuscript. YTL, SD, LZ, and DZ revised the manuscript.

## Conflict of Interest

The authors declare that the research was conducted in the absence of any commercial or financial relationships that could be construed as a potential conflict of interest.

## Footnotes

1 http://drive5.com/uparse/

2 http://qiime.org/scripts/alpha_diversity.html

3 http://qiime.org/scripts/upgma_cluster.html

4 http://ab.inf.uni-tuebingen.de/software/megan4/
