# Supplementary material for: Significant Impacts of Both Total Amount and Availability of Heavy Metals on the Functions and Assembly of Soil Microbial Communities in Different Land Use Patterns
Source: Front Microbiol. 2019 Oct 4;10:2293. doi: 10.3389/fmicb.2019.02293 (PMC6788306; doi:10.3389/fmicb.2019.02293)
Supplement: Supplementary file 1 [file Data_Sheet_1.docx]

**Significant impacts of both** **total amount and availability of heavy metals on the** **functions and assembly of soil microbial communities in different land use patterns**

Zhen Zhen^a, #^, Sibo Wang^b#^, Shuwen Luo^b^, Lei Ren^a,^ Yanqiu Liang^b^, Rongchao Yang^a^, Yongtao Li^c^, Yueqin Zhang^a^, Songqiang Deng^d^, Lina Zou^d^, Zhong Lin^b,*^, Dayi Zhang^e,*^

a. Agriculture College, Guangdong Ocean University, Zhanjiang, 524088, PR China

b. Faculty of Chemistry and Environmental Science, Guangdong Ocean University, Zhanjiang, 524088, PR China

c. College of Natural Resources and Environment, South China Agricultural University, Guangzhou, 510642, PR China

d. Research Institute for Environmental Innovation (Suzhou), Tsinghua, Suzhou 215163, China

e. School of Environment, Tsinghua University, Beijing, 100084, PR China

^#^ Both authors contribute equally to this work.

***Corresponding author**

Dr Dayi Zhang

School of Environment, Tsinghua University, Beijing, 100084, PR China

Tel.: +86(0)62773232; Fax: +86(0)62785687; Email: zhangdayi@tsinghua.edu.cn

Dr Zhong Lin

Faculty of Chemistry and Environmental Science, Guangdong Ocean University, Zhanjiang, 524088, PR China

Tel.: +86 0759 2383311; Fax: +86 0759 2383311; Email: [linzhong@gdou.edu.cn](mailto:linzhong@gdou.edu.cn)

**Table S1.** Latitude and longitude of sampling sites in the studied areas.

| **Site** | **latitude** | **Longitude** |
| --- | --- | --- |
| OF_SM | 24°42'21.10" | 113°38'48.43" |
| OF_CFPP | 24°35'15.42" | 113°34'58.15" |
| OF_SP | 24°43'17.50" | 113°34'29.66" |
| MF_PS | 24°31'16.82" | 113°41'51.15" |
| MF_US | 24°33'25.93" | 113°41'43.84" |
| MF_MS | 24°33'19.10" | 113°42'58.80" |
| FF_P | 24°52'17.90" | 113°32'17.83" |
| FF_E | 24°47'54.65" | 113°35'27.77" |
| FF_S | 25°7'33.25" | 113°21'5.68" |

**Table S2.** Sequencing raw data and α-diversity in different areas.

| **Sample** | **Effective Tags** | **OTU Number** | **Shannon** | **Chao1** | **Faith PD** |
| --- | --- | --- | --- | --- | --- |
| OF_SM | 25281 | 2572 | 6.87 | 5946 | 137.25 |
| OF_CFPP | 29647 | 2757 | 6.86 | 7931 | 149.04 |
| OF_SP | 27945 | 2879 | 6.96 | 6201 | 154.20 |
| MF_PS | 24225 | 2326 | 6.37 | 4519 | 112.74 |
| MF_US | 22772 | 2196 | 5.99 | 4122 | 110.17 |
| MF_MS | 19633 | 1961 | 5.21 | 4382 | 115.65 |
| FF_P | 31158 | 2935 | 7.58 | 6100 | 161.61 |
| FF_E | 36672 | 3263 | 8.42 | 6590 | 162.63 |
| FF_S | 34752 | 3085 | 8.39 | 6855 | 161.11 |


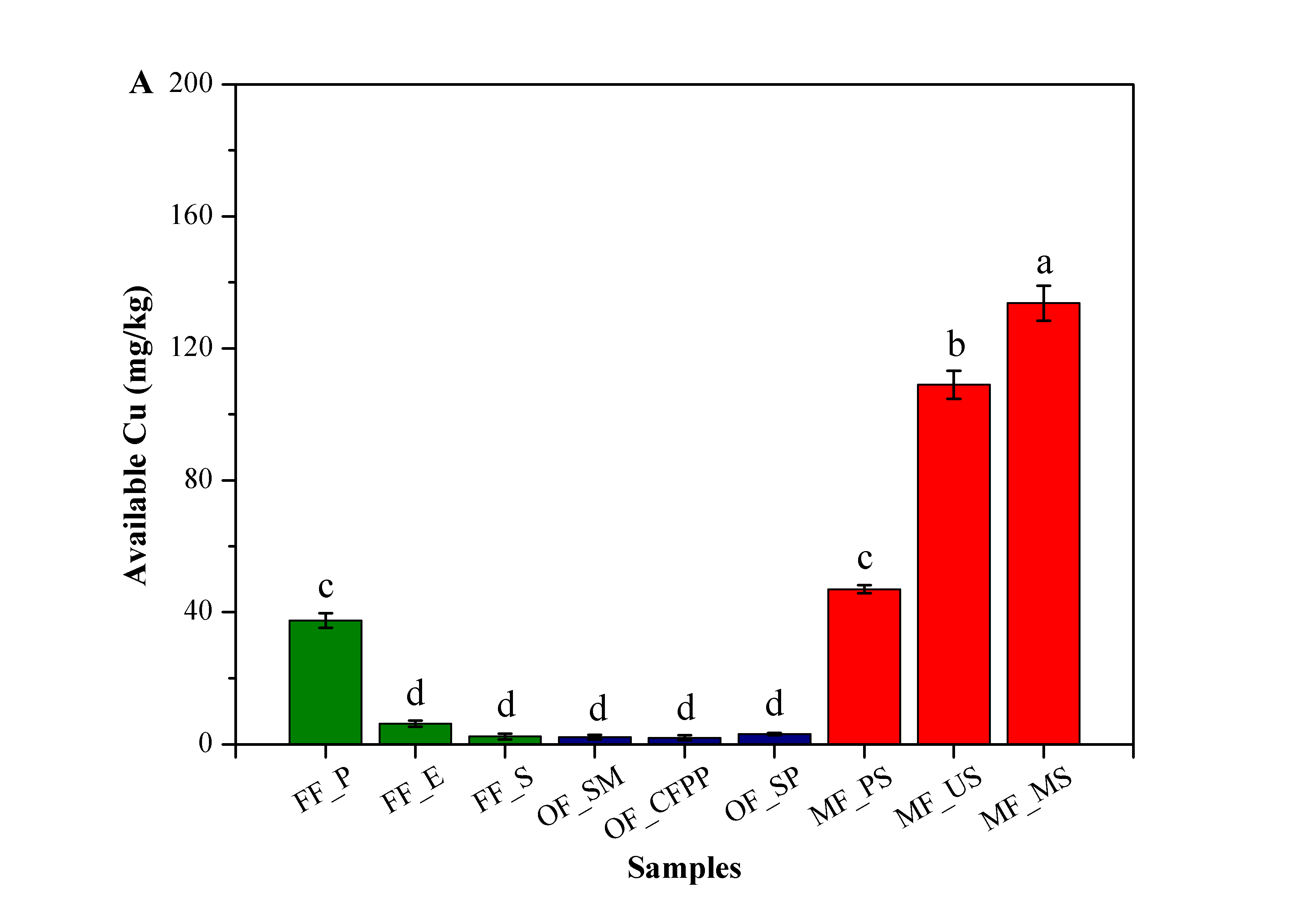

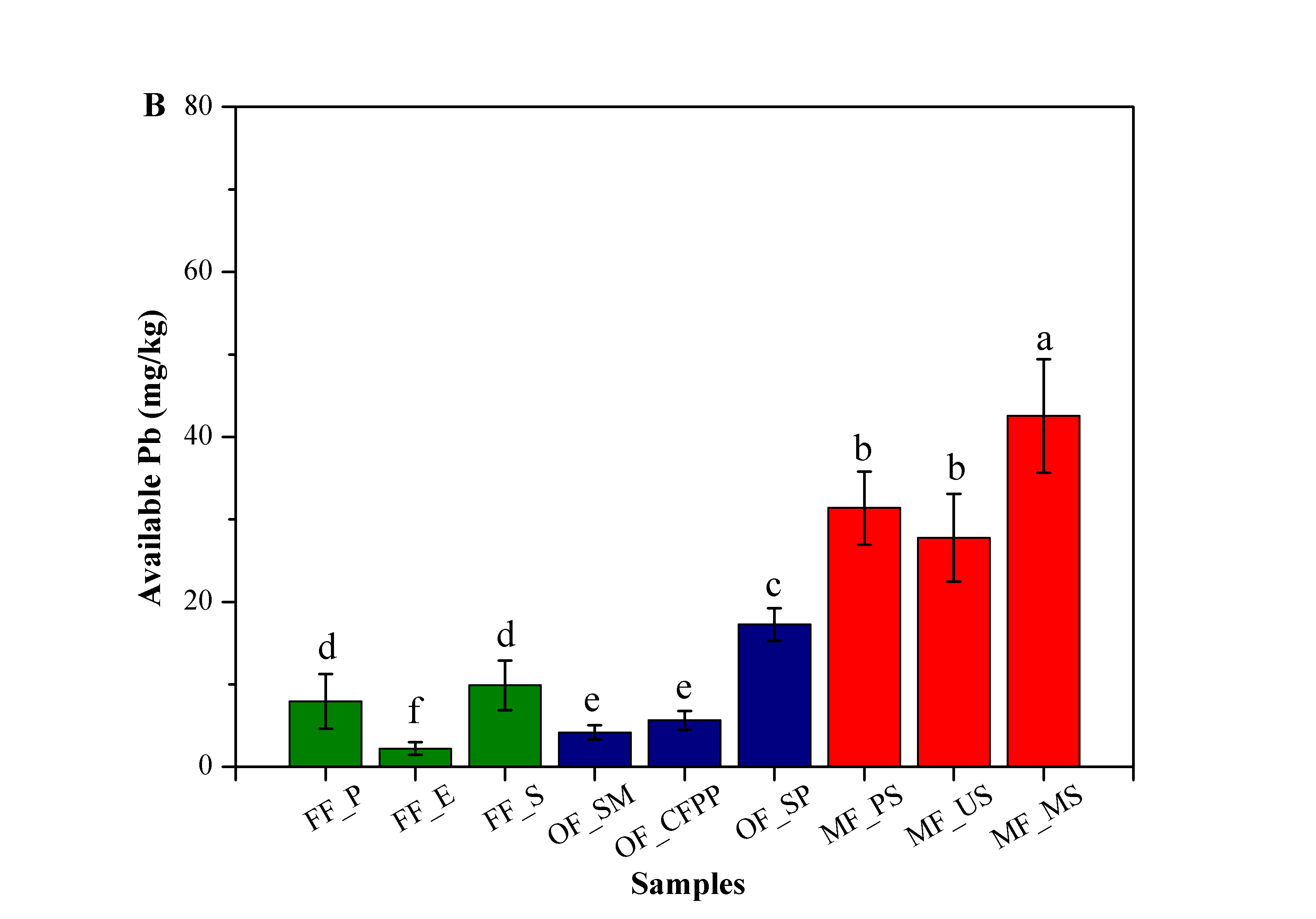

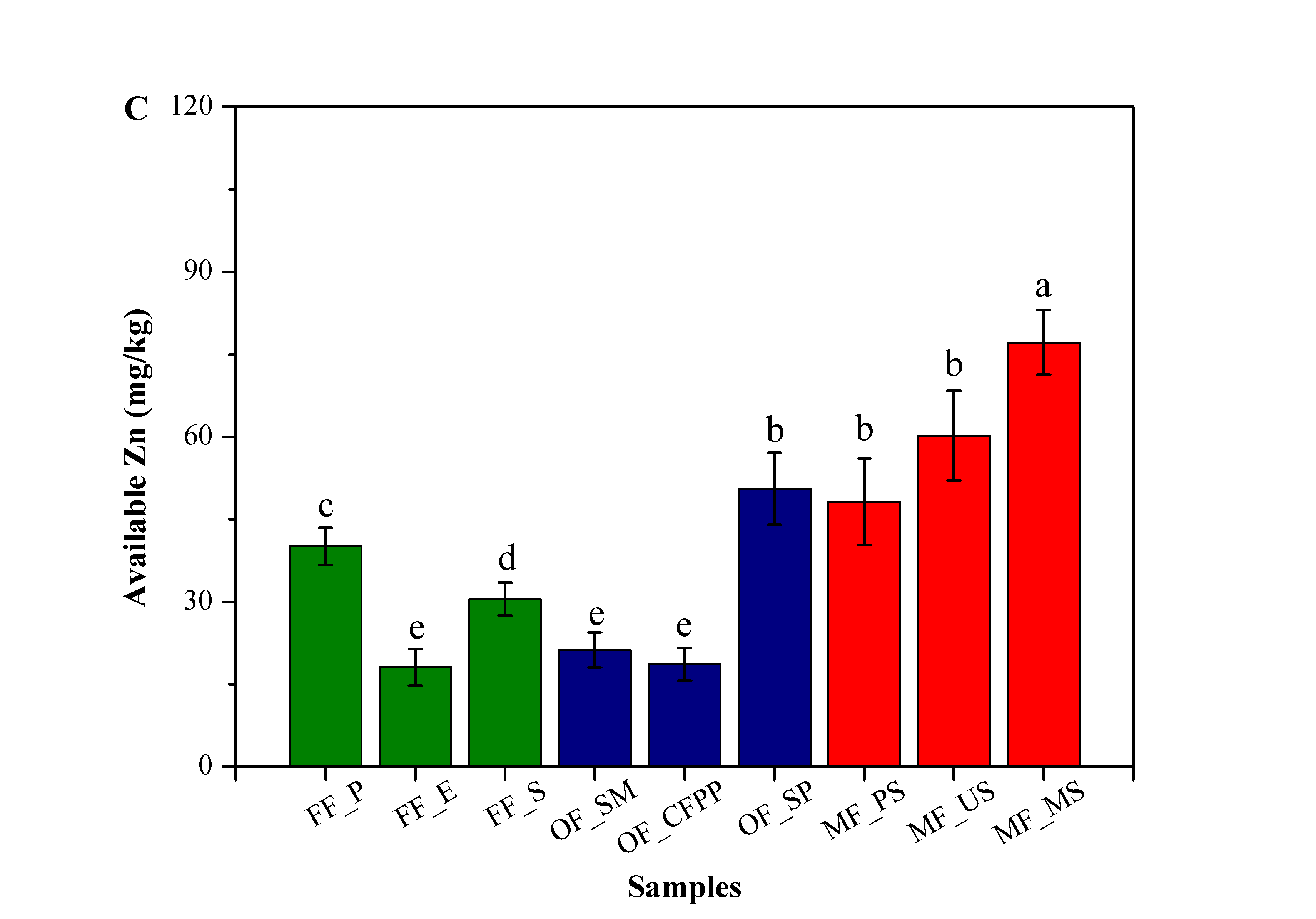

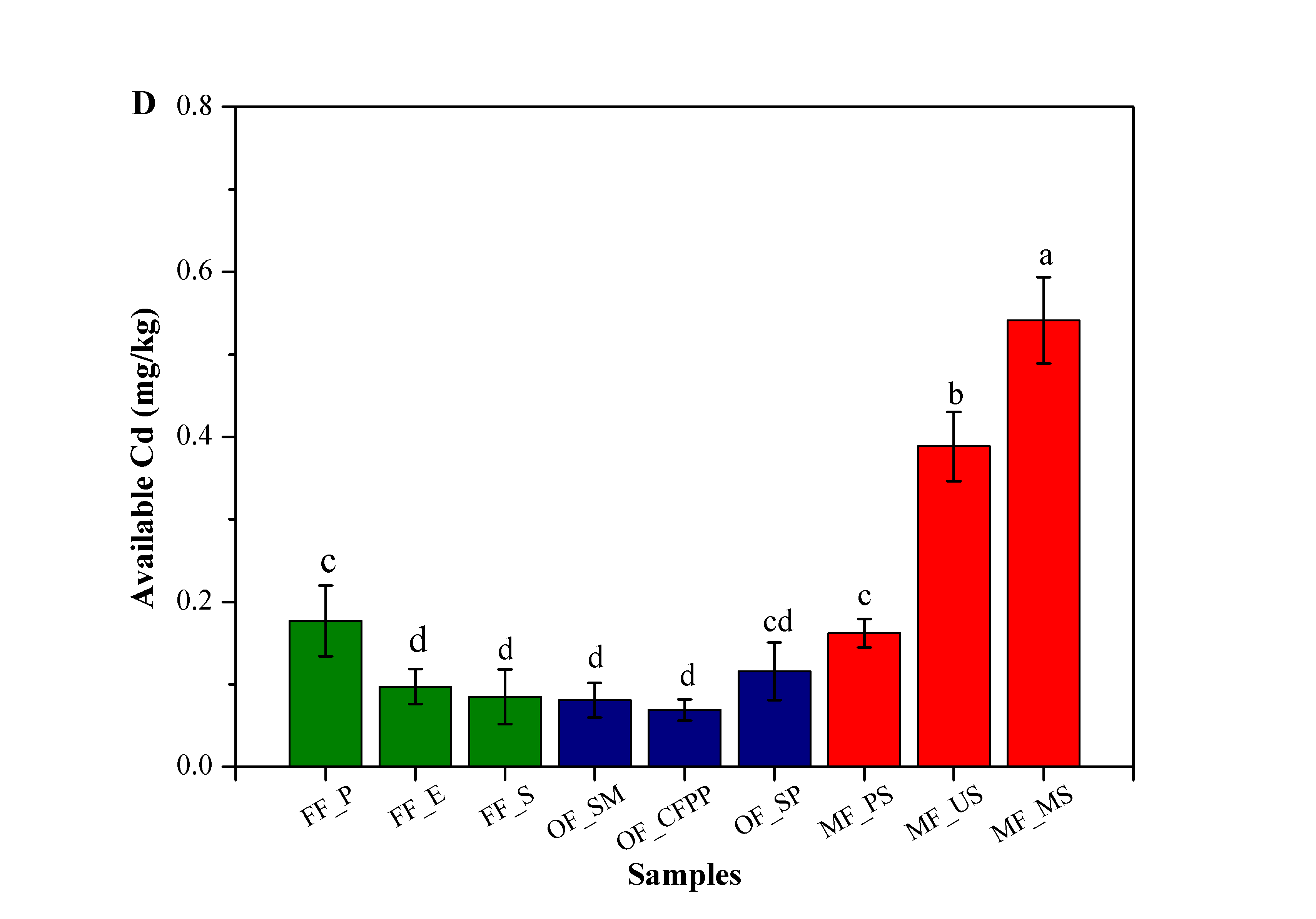

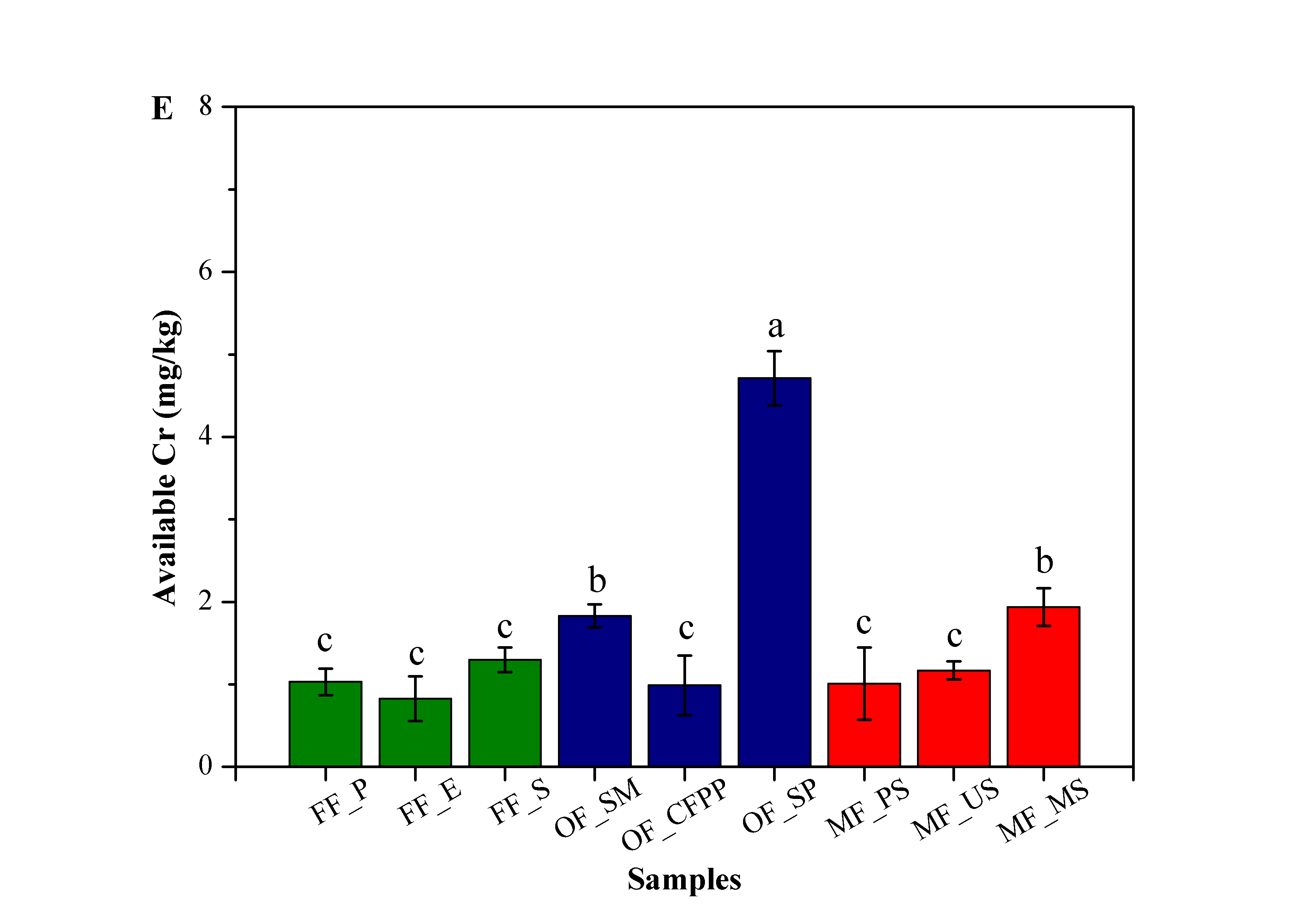

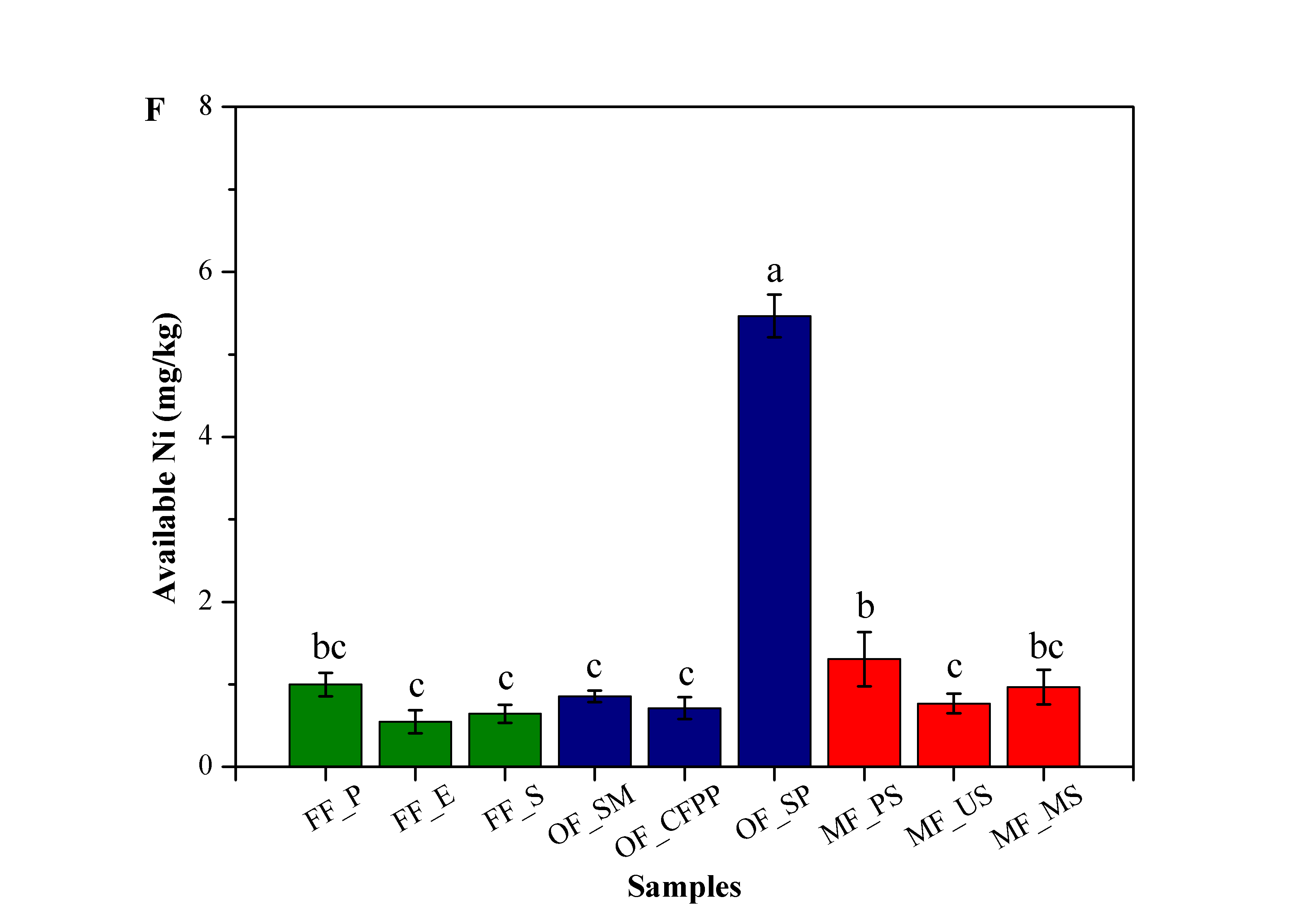


**Figure S1.** Available HMs across different land use patterns. (A) Cu, (B) Pb, (C) Zn, (D) Cd, (E) Cr and (F) Ni. Data are mean ± standard deviation (SD) obtained from five replicates. Bars with different lower-case letters refer to significant differences (ANOVA, Duncan's test, p<0.05) among the samples and the same letter indicates no significant difference.
